# Pollution of water bodies and related impacts on aquatic ecosystems and ecosystem services: The case of Ghana's booming ‘galamsey’ industry

**DOI:** 10.1016/j.heliyon.2024.e40880

**Published:** 2024-12-03

**Authors:** Kenneth Bedu-Addo, Louis Boansi Okofo, Augustine Ntiamoah, Henry Mensah

**Affiliations:** a School of Engineering and Architecture, SRH Hochschule, Heidelberg, Germany; b Aqua Kommunal Services GmbH, Frankfurt Order, Germany; c Department of Chemical Engineering, College of Engineering, Kwame Nkrumah University of Science and Technology, Kumasi, Ghana; d Centre for Settlements Studies, Kwame Nkrumah University of Science and Technology, Kumasi, Ghana

**Keywords:** Galamsey, Aquatic ecosystems and ecosystems services, Driver-pressure-state-impact-response framework, Quantitative defensible impact characterization, Impact significance rating

## Abstract

Galamsey is a Ghanaian jargon from the phrase “gather and sell,” coined from how gold was mined with simple tools by natives and sold afterwards. Despite its socio-economic benefits, a significant upsurge in galamsey has been widely associated with significant environmental impacts viz, destruction of aquatic ecosystems and ecosystems services. This paper discuses impacts of galamsey on aquatic ecosystems and ecosystem services using the Driver-Pressure-State-Impact-Response framework in combination with the quantitative defensible impact characterization approach to establish the cause-and-impact relationships between pollutants associated with galamsey, the extent to which aquatic ecosystems and ecosystem services are impacted while answering the questions what is happening to the environment and why it is happening (compilation and analysis of status and trends of key environmental indicators) and what the consequences are for the environment (analysis of impacts of environmental change on ecosystem services). There were highly significant differences (P < 0001) between the analysed mean Hg, As and turbidity levels and the permissible levels in surface water with the potential of affecting ecosystem services including water for drinking, water for recreational, water for irrigational, and water for aqua culture purposes. Spiritual values, symbolic values, recreation, aqua culture and fish harvest were among ecosystem services highly impacted with a high impact significance ranged 101–125 (++++orange) while aesthetic experience was very highly impacted with a very high impact significance ranged between 126 and 150 (+++++red). Discolouration by silt, and hydromorphological alteration had a medium-high impact significance ranged from 76 to 100 (+++yellow) while spiritual value had a low-medium impact significance ranged from 51 to 75 (represented by ++green). It is recommended that efforts are made to promote responsible cleaner operations in the artisanal and small-scale gold mining sector through the provision of subsidized eco-friendly tools for gold amalgamation in addition to regular capacity building training on the impacts of galamsey on ecosystems, ecosystem services and human health using cycle of cause-effect-outlook relationship.

## 1 Introduction

Although it is extremely difficult to state the exact date humans ventured into gold exploration, since its discovery, the aura around gold has captivated humans of all cultures globally. Gold's everlasting gleam, unchanging uniqueness, unfading reflective sunlit lustre, and its comparison to the sun's perpetuity have until contemporary times been the epithet of its symbolic and revered statues for which humans have always craved [1,2]. In order to satisfy man's insatiable desire for gold, humans used rudimentary tools, in one of the most important industries of early civilization [3]. Present day Ghana, a country located in West Africa, was not left out of the countries that saw gold mining as a viable economic venture for which reason she was mining gold long before the trans-Saharan trade. The abundance of gold earned present day Ghana its colonial name Gold Coast [4].

Before the European explorers arrived on the shores of present-day Ghana in 1471, artisanal gold mining was a major industry that supported the Akan speaking states. Traditional gold exploitation techniques, which were rudimentary in nature with minimal environmental impacts, constituted a highly respected traditional vocation in the then Gold Coast. Groups, families and individuals were the main custodians and owners of parcels of land enriched with gold ore in their communities [5,6].

The arrival of the European explorers in then Gold Coast, which saw them introduce large-scale gold mining after colonization, marked the beginning of groups, families and individuals who hitherto were custodians of gold-rich land being muscled out [6]. To restore normalcy, appease traditional authorities, create jobs, and propel the economy, the ‘’Small-scale Gold Mining Law, 1989 (PNDC Law 218)’’ which officially legalised artisanal and small-scale mining in Ghana was passed with much hope and economic expectations three decades after Ghana gained independence from British rule [7]. This joy was however, short-lived due to bureaucracies associated with the acquisition of mining concessions and licences under the very law that gave the groups, families, individuals and traditional authorities so much hope (Hilson, 2020; [[8], [9], [10]]).

Driven by an inherent will to survive, many inhabitants of rural communities in Ghana took to ‘galamsey’ a Ghanaian jargon from the phrase “gather and sell,” coined from observation of how gold was mined with simple tools by natives and sold afterwards. to circumvent the unending bureaucracies associated with the acquisition of mining concessions and licences under ‘’The Small-scale Gold Mining Law, 1989, PNDC Law 218’’ [11]. This drive towards economic survival saw the artisanal and small-scale mining (ASM) sector (both legal and galamsey) in Ghana contribute a significant 43 % of the total gold produced in 2018. However, this significantly high contribution by the ASM sector comes at a huge environmental and public health cost. The use of heavy earth moving equipment for excavation, which has replaced the use of rudimentary techniques at some galamsey sites, has resulted in massive destruction of forest reserves, fertile agricultural lands, and vegetation cover in Ghana [12,13].

**Fig. 1:**
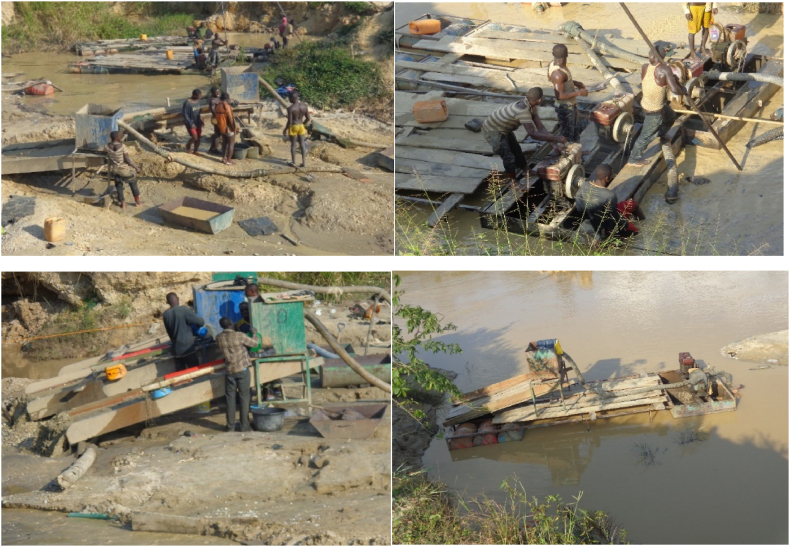
Impacts of galamsey on River Offin; Photo Credit: Kenneth Bedu-Addo.

Galamsey activities in the Ashanti Region, Eastern and Western Regions of Ghana has led to massive loss of forest cover resulting in loss of aquatic and terrestrial habitats affecting the distribution of flora and fauna ultimately distorting the ecological structures of local communities. In freshwater aquatic environments such as the Pra, Offin, Ankobra and Birim Rivers, mercury which is predominately used by artisanal miners during amalgamation of gold is transformed into methyl mercury, an extremely toxic species of mercury with a huge potential of bioaccumulating in aquatic organisms and biomagnifying along aquatic food chains ([14,15]; Obeng et al., 2019 [16,17]; Gilbert and Osei-Bonsu, 2016). Additionally, colliery effluents high in fine and coarse particles associated with galamsey activities all over Ghana has turned pristine clear water bodies muddy impacting negatively on aquatic ecosystems (Fig. 1).

The muddy slurry effluent from galamsey sites has reduced sunlight penetrability in water bodies seriously affecting primary productivity, habitats of indigenous traditional fish species as well as the functionality and integrity of freshwater ecosystems ([[18], [19], [20], [21], [22], [23]]; Kemker; 2014; [24]). United Nations [25] assertion of sparse water quality in African countries is a signal aquatic ecosystem restoration as a tool for the improvement of access to ecosystem services will continue to be a daunting task [26]. The assertion by Ref. [25] corroborates the paucity of research on the impacts of ‘galamsey’ on ecosystem services viz provision, regulatory and cultural ecosystem services in Ghana. This article seeks to fill the gap by using the Drivers-Pressures-State-Impact-Response (DPSIR) framework proposed by UNEP in tandem with the quantitative, defensible impact characterization approach to find answers to two key questions namely: (i) what is happening to aquatic environment because of galamsey and why this is happening? (*compilation and analysis of status and trends of key environmental indicators);* and (ii) what the consequences are for the aquatic environment on the provision, regulatory and cultural ecosystem services? (*analysis of impacts of environmental change on human health and ecosystem services).*

## 2 Location, climate and physical features

### 2.1 Country snapshot

Ghana is located in the West Coast of Africa with its southernmost point five degrees north of the Equator with the Greenwich meridian passing through its industrial city of Tema. With geographic coordinates of 8 00 N, 2 00, Ghana has a 539 km coastline along the Gulf of Guinea and shares borders to the east with Togo, north with Burkina Faso, to the west with Côte d’Ivoire and to the south with the Gulf of Guinea. Ghana's climate is generally tropical with the northern enclave being hot and dry, the southwestern enclave being hot and humid and the south-eastern enclave being dry. The southern enclaves are characterised by two rainy seasons, a minor season between September–November and a major season between April–July attaining a maximum in June. Mean annual rainfall figures in these areas range between 1250 and 2150 mm. The climate in the northern enclave is predominantly semi-arid, which is influenced by the movement of the inter tropical convergence zone (ITCZ). The ITCZ brings cool dry Northeast Trade winds (Harmattan) in the dry season between November and March, and moist Southwest Moonson in the wet season between April and September. The rainy season is unimodal with an average annual rainfall value of 980 mm/year ([27], 20121).

**Fig. 2:**
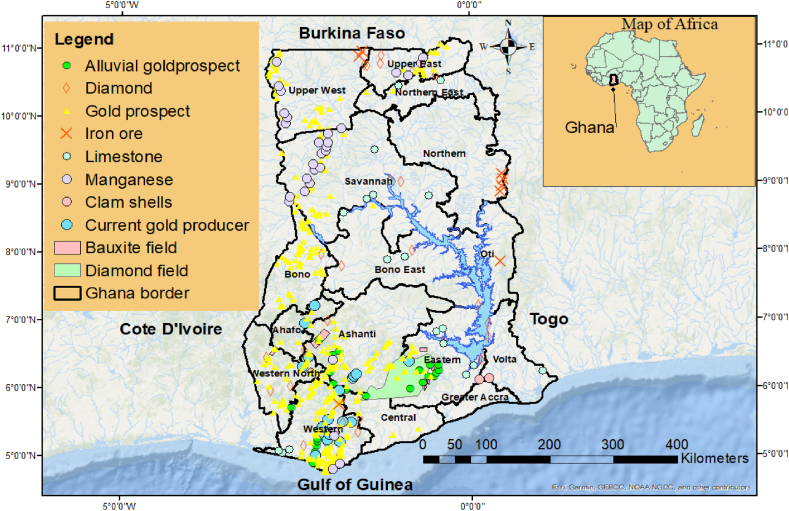
Mineral distribution and prospects in Ghana.

Currently, Ghana hosts a number of the world's gold hulks including Xtra Gold, Newmont Gold Ltd, Perseus Mining, Gold Fields Ghana, AngloGold Ashanti and Golden Star among others (Mineral Commission of Ghana, 2023). The gold deposits are hosted in two main geological formations (Fig. 2) the metasedimentary and metavolcanic rocks associated with mesothermal quartz-vein gold Deposits and conglomerate rocks associated with paleoplacer gold deposits. Alluvial gold deposits can also be found in the metasediments along some rivers in Ghana [28].

## 3 Materials and methods

**Fig. 3:**
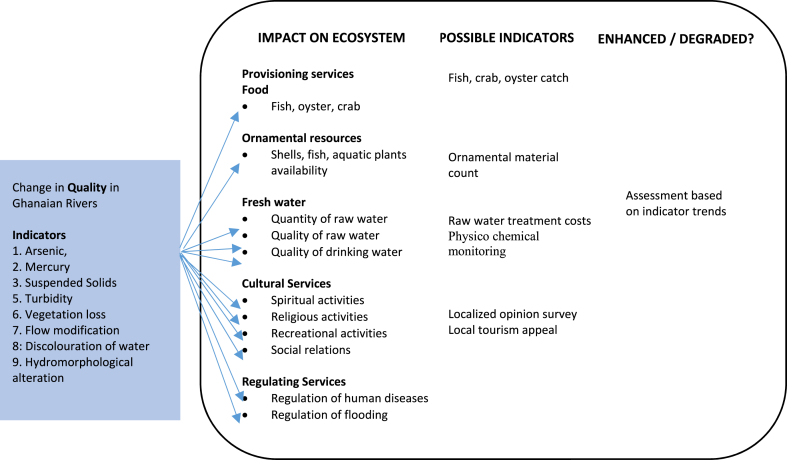
Impact pathway assessment indicators (adapted from Ref. [29]).

An analysis of the impacts of galamsey on ecosystems and ecosystem services in Ghana was undertaken based on a three (3) step analytical approach adapted from UNEP's human Environment analytical approach. The first of the three steps entailed a pre-assessment stage, during which an indicator impact pathway diagram (Fig. 3) was developed during four (4) experts' interviews, site visits, review of documents a comprehensive desk study based on five (5) thematic areas namely impacts of galamsey on aquatic ecosystems, impacts of galamsey on aquatic ecosystem provision services, impacts of galamsey on aquatic ecosystem regulatory services, impacts of galamsey on aquatic ecosystem cultural services, and impacts of galamsey on human health. Secondary data sources on the five thematic areas used in the study included peer reviewed and non-peer reviewed journals, books, institutional publications, online databases including Google Scholar, online blogs, case studies, and articles from credible sources. An inclusion criteria taking into cognizance the reputation of the source of information, bias of information due to interest from a sponsor, corroboration of information from other sources, built-in credibility of information as well as credible academic journals and publishers was used to gather data for answering the questions what is happening to the aquatic environments in Ghana and why it was happening? (*compilation and analysis of status and trends of key environmental indicators) and* what the consequences are for the environment and people? (*analysis of impacts of environmental change on ecosystem services.*

The desk study then formed the basis for the use of impact pathway diagram (Fig. 3) with indicators namely vegetation loss, hydromorphological alteration, flow modification, discolouration of water, arsenic, mercury, suspended solids, and turbidity levels for the initial impact assessment of galamsey activities on aquatic ecosystems and the ecosystem services they provide. Arsenic was selected as an indicator for the study because the gold ore in the Dunkwa-On-Offin area is embedded in arsenopyrite which leads to the release of arsenic during galamsey activities. Mercury was selected as an indicator by virtue of its use by the galamsey miners for amalgamation purposes with water bodies serving as receptacles for unrecovered mercury. The activities of galamsey miners in the study area which is mainly alluvial generates huge quantities of mud during the mining and recovery processes there altering the suspended solids loads, colour and turbidity of water bodies in Ghana (Fig. 1). The huge quantities of mud generated during galamsey activities in tandem with the diversion of rivers are precursors for flooding of arable land, river flow modification, hydromorphological alteration well as vegetation loss (Fig. 3).

**Fig. 4:**
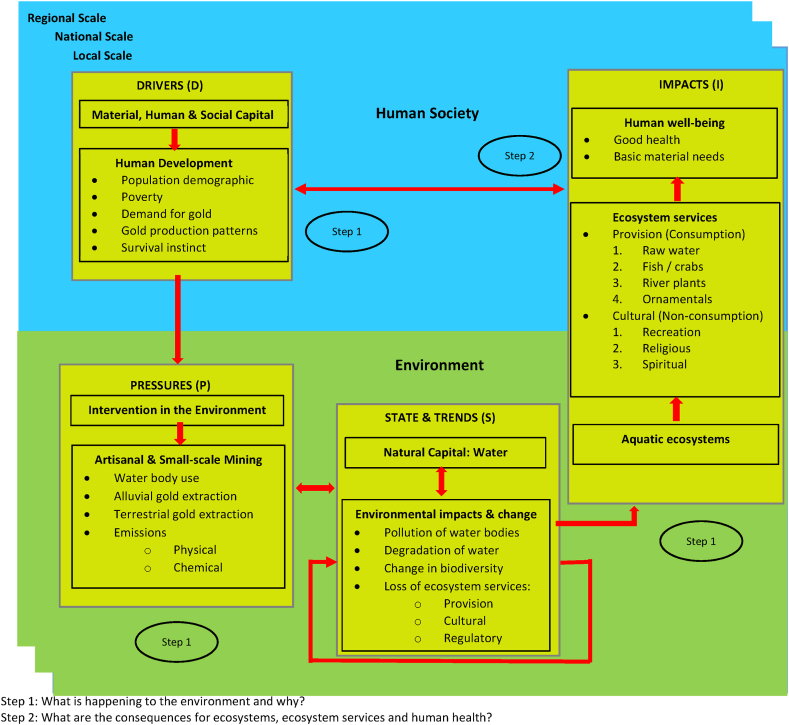
Adapted D-P-S-I framework based on the UNEP human environment approach.

Source: Adapted [30]

The indicators were further used in an adapted version of the DPSIR analytical framework namely DPSI (Fig. 4) in combination with the quantitative, defensible impact characterization approach (Table 1)

**Table 1:**
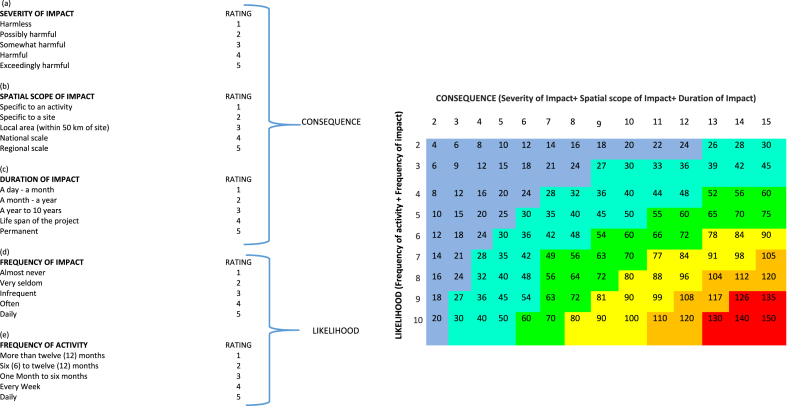
Quantitative, defensible impact characterization for the assessment of significance of impacts attributable to galamsey.

which was selected based on its credibility as a recommended Training Resource Manual on Environmental Impact Assessment by the United Nations Environmental Programme and the experience of the authors on impact assessment to find answerers to two key questions (i) what is happening to the aquatic environments in Ghana and why it was happening? (*compilation and analysis of status and trends of key environmental indicators) and (ii)* what the consequences are for the environment and people? (*analysis of impacts of environmental change on human health and ecosystem services.* In answering the question what is happening to the aquatic environments in Ghana and why it was happening, the levels of mercury and arsenic in river Offin a galamsey impacted water body was undertaken by taking 500 ml snap water over a 1000m distance in clean labelled sampling bottles thoroughly rinsed with distilled water and pre-conditioned with Nitric acid (HNO3) to keep the integrity of the sample for arsenic and mercury analysis. Five snap samples which were taken 200m apart taking into cognizance variation in depth of sampling, sampling site history, field observations, uniformity of the sampling point was thorough mix to give representative composite sample of River Offin over a 1000m stretch. The composite sample generated out of the five snap samples was placed in a cool box with ice packs and sent to the lab for the determination of mercury and arsenic concentrations. The 1000 ml of the composite water sample was filtered through a 0.45 μm filter after which 200 ml was analysed with a Varian A220 Flame Atomic Absorption Spectrometer at wavelengths of 253.7 nm and 193.7 nm for Hg and As respectively. Turbidity readings were done on site using the HI97727 colour of water photometer which has an advanced optical system to provides a narrow band interference filter to ensure accurate readings. A certified reference material for Hg, As and turbidity was prepared and analysed for quality assurance purposes. The readings obtained from the lab analysis using the HI97727 colour of water photometer and the Varian A220 Flame Atomic Absorption Spectrometer were subjected to Student T-Test analysis in GraphPad Prism 7 to ascertain significance between the permissible levels of As, Hg and turbidity and the measured values for As, Hg and turbidity in the analysed water samples.

**Fig. 5:**
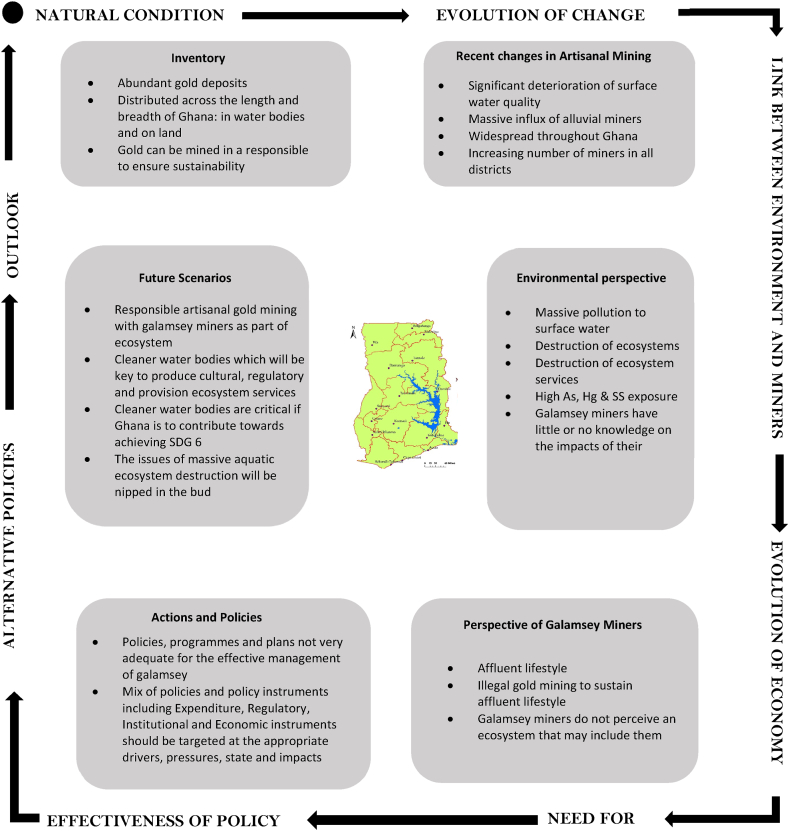
Cycle of cause-effect-outlook relationship for responsible artisanal mining in Ghana.

To answer the question what the consequences of galamsey are for the aquatic environments and the people of Ghana (*analysis of impacts of environmental change on human health and ecosystem services*), the indicator impact pathway was used. Each indicator was rated based on *severity of impact + spatial scope of impact + duration of impact* (consequence of impact with an upper limit value of 15) and *frequency of impact + frequency of activity* (likelihood of the impact with a maximum value of 10). Impact significance attributable to galamsey activities was finally derived as shown in the rating matrix (Table 1). The various colours in the rating matrix were interpreted as follows: a very high impact significance ranged between 126 and 150 (+++++red), a high impact significance ranged 101–125 (++++orange), a medium-high impact significance ranged from 76 to 100 (+++yellow), low-medium impact significance ranged from 51 to 75 (++green), a low impact significance ranged from 26 to 50 (+teal), an extremely low or no impact significance ranged from 1 to 25 (0blue). A cycle of cause-effect-outlook relationship for responsible artisanal mining in Ghana (Fig. 5) which had artisanal miners as part of the ecosystem was developed as a recommendation towards responsible mining at the artisanal and small-scale level in Ghana.

## 4 Results and discussion

Table 2 presents a comparison between the mean levels of arsenic, mercury, and turbidity in galamsey polluted water bodies and the Ghana Environmental Protection Agency (EPA) permissible levels of arsenic, mercury, and turbidity for surface water. There were highly significant differences (P < 0001) between the mean Hg, As and turbidity levels with the potential of affecting ecosystem services.

**Table 2:** Comparison of levels of arsenic, mercury, turbidity, and permissible discharge levels in galamsey impacted rivers in the context of ecosystem services.

Ecosystem Service	Arsenic Conc. (mg/L)		P-value	Mercury Conc. (mg/L)		P-value	Turbity (NTU)		P-value
	Mean	Stand		Mean	Stand		Mean	Stand	
**Drinking (Provision)**	35.36	0.025	P < 0.0001	87.5	0.001	P < 0.0001	1600	1	P < 0.0001
**Recreation (Cultural)**	35.36	0.05	P < 0.0001	87.5	0.001	P < 0.0001	1600	50	P < 0.0001
**Irrigation (Provision)**	35.36	0.01	P < 0.0001	87.5	–		1600	–	
**Aqua culture (Provision)**	35.36	0.005	P < 0.0001	87.5	0.001	P < 0.0001	1600	25	P < 0.0001

P is significant at p < 0.05; Stand = Permissible discharge level; Conc. = Concentration; NTU=Nephelometric turbidity unit.

Table 3 shows significance of impacts attributable to gold mining operations assessed via rating each parameter under consequence of the impact with a maximum value of fifteen (15) and likelihood of the impact with a maximum value of ten (10). The product of consequence and likelihood of 140 and 42 are interpreted as having very high significance and low significance respectively. Mercury and loss of vegetation had the highest and least significance ratings.

**Table 3:** Significance rating for galamsey related indicators based on severity of impact, spatial scope of impact and duration of impact.

	Arsenic	Suspended Solids	Vegetation Loss	Flow Modification	Hydromorphological Alteration	Discolouration	Erosion	Mercury
Severity of Impact	5	4	2	3	3	2	3	5
Spatial Scope of Impact	3	4	2	2	2	4	3	5
Duration of Impact	4	4	2	5	5	4	3	4

**Consequence of Impact (C)**	**12**	**12**	**6**	**10**	**10**	**10**	**9**	**14**

Frequency of Impact	4	5	2	3	3	4	3	5
Frequency of Activity	5	5	5	5	5	5	5	5

**Likelihood of Impact (L)**	**9**	**10**	**6**	**8**	**8**	**9**	**8**	**10**

Significance of Impact (Consequence x Likelihood)	**High (12x9 = 108)**	**High (12x10 = 120)**	**Low (6x7 = 42)**	**Medium-high (10x8 = 80)**	**Medium-high (10x8 = 80)**	**Medium-high (10x9 = 90)**	**Low-medium (9x8 = 72)**	**Very high (14x10 = 140)**

Table 4 presents the results of impact significance matrix of galamsey on ecosystem provision services using the *severity of impact, spatial scope of impact, duration of impact***,** *frequency of impact and frequency of activity* using arsenic, mercury suspended solids, discolouration of Water, erosion, flow modification and hydromorphological alteration s indicators. A very high impact significance ranges between 126 and 150 (+++++ red), a high impact significance ranges 101–125 (++++orange), a medium-high impact significance ranges from 76 to 100 (+++yellow), low-medium impact significance ranges from 51 to 75 (++green), a low impact significance ranges from 26 to 50 (+teal), an extremely low or no impact significance ranges from 1 to 25 (0blue).

**Table 4:**
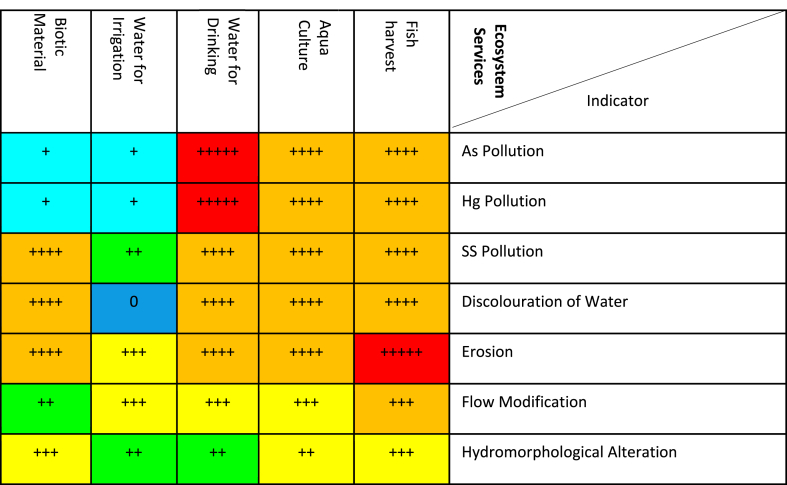
Impacts of galamsey activities on provision ecosystem services in Ghana.

Table 5 is presented as results of impact significance matrix of galamsey on cultural ecosystem services using the *severity of impact, spatial scope of impact, duration of impact***,** *frequency of impact and frequency of activity* using arsenic, mercury suspended solids, discolouration of Water, erosion, flow modification and hydromorphological alteration s indicators. A very high impact significance ranges between 126 and 150 (+++++ red), a high impact significance ranges 101–125 (++++orange), a medium-high impact significance ranges from 76 to 100 (+++yellow), low-medium impact significance ranges from 51 to 75 (++green), a low impact significance ranges from 26 to 50 (+teal), an extremely low or no impact significance ranges from 1 to 25 (0blue).

**Table 5:**
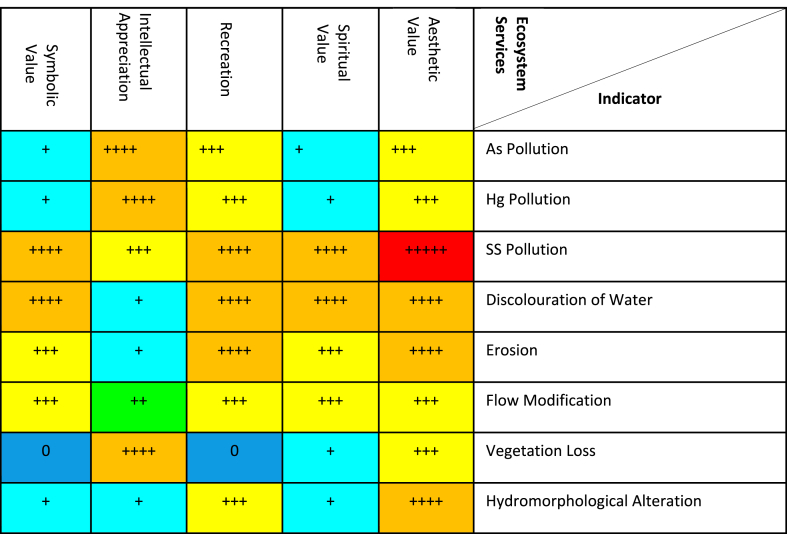
Impacts of galamsey activities on cultural ecosystem services in Ghana.

Table 6 is presented as results of impact significance matrix of galamsey on regulatory ecosystem services using the *severity of impact, spatial scope of impact, duration of impact***,** *frequency of impact and frequency of activity* using arsenic, mercury suspended solids, discolouration of Water, erosion, flow modification and hydromorphological alteration s indicators. A very high impact significance ranges between 126 and 150 (+++++ red), a high impact significance ranges 101–125 (++++orange), a medium-high impact significance ranges from 76 to 100 (+++yellow), low-medium impact significance ranges from 51 to 75 (++green), a low impact significance ranges from 26 to 50 (+teal), an extremely low or no impact significance ranges from 1 to 25 (0blue).

**Table 6:**
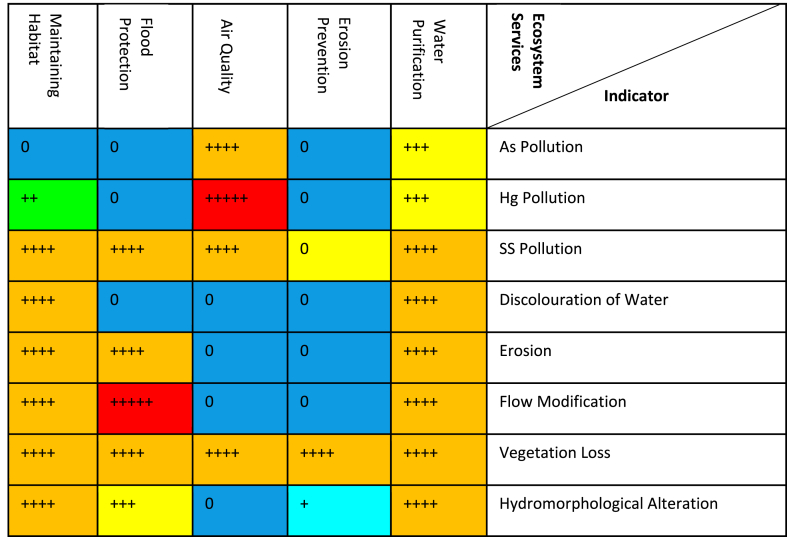
Impacts of galamsey activities on regulatory ecosystem services in Ghana.

## 5 Ecological impacts galamsey in Ghana

Prior to the advent of galamsey, Ghanaian residents in towns and villages have mined gold at the artisanal level for economic reasons in a responsible and sustainable manner using rudimentary tools for centuries. Current divers identified as increasing youth unemployment, poverty, non-enforcement of ACT 703, rising gold prices and a slump in agriculture has led to an escalation of galamsey activities over the last few years across the length and breadth of Ghana [9,24,[31], [32], [33]]. Gamlasey has thus largely replaced subsistence agriculture, which hitherto was the principal income-earning activity in most Ghanaian communities. The galamsey miners operate without valid licenses from the Minerals Commission of Ghana and undertake their mining activities regardless the Water Resources Commission of Ghana's vision of ‘Sustainable water management by all for all’ resulting in debilitating aquatic ecosystems impacts. The impacts caused by galamsey activities in Ghana include altering of predation behaviours, altering of light penetration in water bodies, destruction of aquatic habitats, fragmentation/loss of aquatic habitats and destruction of aquatic ecosystems, which may lead to the loss of aquatic biodiversity as, is evidence at Tontokrom, Kyekyewere and Tarkwa Nsuam in in in the Ashanti, Central and Western Regions of Ghana.

Galamsey activities across the length and breadth of Ghana has and could lead to the diversion of river flow, cause mercury pollution and siltation ([20,34,35]; Hilson et al., 2014; [24]). The mercury used during the amalgamation process of gold recovery by the galamseyers could be deposited in sediments of aquatic systems where it could be converted to methylmercury by microorganisms and absorbed by phytoplankton making it available for accumulation in consumers along the food chains. The mercury could also undergo bioaccumulation in fish, snails, crabs and biomagnify along food chains and food webs thus making galamsey one of the leading contributors to mercury pollution in Ghana (UN Environment, 2019; [[36], [37], [38]]).

The diversion of rivers by galamsey miners in Ghana could lead to the flooding of arable land and the transport of colliery effluent in water bodies negatively affecting the sink capacity of the water bodies and suitability of the water bodies to serve as a habitat for aquatic organisms. The colliery effluents from galamsey activities which are normally high in fine and coarse particles has turned once pristine clear water bodies including the Oda, Ankobra, Pra, Birim and Offin rivers into various shades of brown rivers in Ghana. The slurry and mud coupled with the unrecovered mercury used by galamseyers for amalgamation purposes during gold recovery has the potential of reducing the penetrability of light in water bodies in Ghana reducing the rate of photosynthesis, inhibiting the growth of aquatic plants, bioaccumulating in several species of fish and other hydrobionts that are crucial to aquatic ecosystem function [18,20]. The silt, sand and clay, which constitute the main suspended solids generated during galamsey, could also darken water bodies, clog the gills of fish, reduce the spawning sites of fish by settling in cracks/crevices, and reduce the hunting ability of predatory aquatic organisms that rely heavily on vision ultimately affecting food chains. Suspended solids generated in huge quantities during galamsey activities could additionally negatively affect the reproduction and health of certain fish species, which rely on visual cues during mating and reproduction. The huge volumes of suspended solids generated during galamsey activities could affect the habitats of traditional and indigenous fish species with the functionality and integrity of aquatic ecosystems in Ghana ultimately being threatened ([18,23]; Kemker; 2014).

## 6 Impacts of galamsey on ecosystem services in Ghana

The impacts of galamsey on ecosystem services is so massive in Ghana one begins to wonder if the economic benefits from galamsey is worth the massive siltation, sedimentation and discolouration of several rivers across Ghana. The negative impacts on galamsey on ecosystem services in Ghana is evident in arsenic pollution, mercury pollution, suspended solids pollution, discolouration of water bodies, flow modification, and hydromorphological alteration of several water bodies [20,39,40]. The extremely high levels of total suspended solids in galamsey related water bodies with its resultant significant p-values between monitored data and permissible levels (P < 0.0001; Table 2) is an indication livelihood from ecosystems services could severely be affected by galamsey [41,42]. The excessive suspended solids loads could impact negatively on key provision services not limited to water being used for navigation, raw water for aqua culture, fish as a food source and raw water for drinking purposes [18]. High-suspended solids load generated by the galamsey activities can block the sun's rays from reaching submerged aquatic plants, which serve as producers in an aquatic ecosystem reducing primary productivity [23]. Additionally, the colliery effluent emanating from galamsey with its associated high levels of coarse and fine suspended solids have the potential of darkening waterbodies in Ghana. The darkening of the water bodies could make the water bodies absorb more heat thereby increasing the temperature of the water bodies. An increase in temperature will decrease dissolved oxygen concentrations due to the higher affinity of suspended solids to sunlight as compared to water molecules. As the heat dissipates to the surrounding water by conduction dissolved, oxygen levels will drop considerable causing stratification and the destruction of hydrobionts some of which are important protein sources for humans [23,[43], [44], [45], [46]].

Provision services, which are the most obvious of the services, provided by ecosystems is most impacted by galamsey activities in Ghana. Among the aquatic ecosystems provision services negatively affected by gold mining in Ghana are fresh water for domestic use, fishes and aquatic resources. Several rivers including the Ankobra, Offin, Anikoko, Tano, Bodwire, Asesree, Assaman, Birim, Pra and Oda some of which are very important intake points for raw water for treatment and distribution to consumers have all seen an escalation in turbidity in recent times. The Kibi, Daboase and Odaso treatment plant treatment plants of the Ghana Water Company Limited had to suspend operations due to extremely high turbidity values of 1600 NTU, 1261 NTU, up to 2000 NTU and 3842 NTU respectively because of sprawling galamsey activities. This could negatively impact on raw water sources an important service humans obtain from aquatic ecosystems. A vast range of aquatic foods including snails, fish and crustaceans (provision service) obtained from aquatic ecosystems are being lost to overuse of water bodies and conversion of same to mining hot spots galamseyers (IPBES, 2019) Additionally, genes and genetic information very critical for aquatic flora and fauna breeding, and biotechnology purposes are being lost due to the destruction of aquatic ecosystems by galamsey activities.

Among the cultural ecosystem services that could be affected by galamsey in Ghana are spiritual values, religious values, educational values, cultural heritage values, recreation, and aesthetic experience [47]. Water bodies desecrated by suspended solids pollution, discolouration by silt, and hydromorphological alteration by galamsey deprive communities of religious values including the use of water bodies for Christian programmes such as baptism and spiritual values including visitations by traditionalist to deities in water bodies to reverse curses among others. Educational values not limited to traditional knowledge systems not limited to visiting river bodies only on specific days to help water bodies self-cleanse and replenish fish stock to meet the protein requirements of inhabitants of communities in Ghana is fast disappearing due to the galamsey activities. The aesthetic and recreation benefits humans obtain from aquatic ecosystems could be totally lost if suspended solids pollution, discolouration of water bodies with silt, and hydromorphological alteration of water bodies attributable to galamsey goes on unabated. A summary of the ecosystem services likely to be negative affected in Ghana as a result of the pollution of water bodies by galamsey activities is presented in Table 7.

**Table 7:** Summary of ecosystem services to be affected in Ghana because galamsey.

Category	Service	Impact of Galamsey on Service
Provisioning	Food	• Reduced fish, oyster and snail catch • Reduced quantities of fish as source of protein • Reduced availability of raw water for aqua culture
	Raw water	• Reduced raw water availability • Reduced raw water quality • Increased raw water treatment cost
	Genetic and ornamental resources	• Reduced and or loss of critical genetic material for aquatic animal and plant breeding • Loss of shells used for making ornaments • Loss of exotic and ornamental fish used in aquarium
	Natural chemicals & pharmaceuticals	• Loss of biocides, traditional medicines and food additives

Regulation	Water regulation and purification	• Increased incidence and magnitude of flooding • Reduced aquifer recharge • Reduced ability of water bodies to self-cleanse
	Erosion control	• Reduced soil retention ability of terrestrial environments due to clearing of vegetation
	Regulation of human diseases	• Increased abundance of diseases causing vectors as a result of the destruction of the habitat of fishes that feed on the larvae of vectors, such as mosquitoes

Cultural	Spiritual and religious and cultural heritage values	• Reduced use of water bodies for religious and spiritual activities not limited to baptism and revoking curse • Loss of identity because of the importance rural communities place on deities' and culturally significant species all of whom dwell in water bodies.
	Aesthetics, recreation and ecotourism	• Decreased use of water bodies for recreation, aesthetic and ecotourism purposes.

## 7 Determination of impacts of galamsey based on quantitative defensible impact characterization

The significantly high concentration of arsenic in water sampled in comparison to the permissible levels P < 0.0001 (Table 2) is expected to have a high significance rating based on the quantitative, defensible impact characterization (Table 3, Table 4). This is because the arsenic concentration exceeded the permissible levels in water bodies in Ghana by over 1400 times (Table 2). Additionally arsenic a highly hazardous inorganic micropollutant of priority can cause hydrobionts toxicity, affect fish growth, fish behaviour and or reproduction in fish. The high significance rating of arsenic is also attributed arsenic's ability to destroy habitats of fish, aquatic mammals, birds, and invertebrates. Arsenic's high significance rating could be attributed to the ability of arsenic to go into soil solution subsequently leaching into ground water aquifers ([34,48]; Liu et al., 2015; [49,50]). Arsenic could have a high impact (++++) on aqua culture and fish harvest due to arsenic’ ability to destroy fish habitat, affect fish growth, behaviour and or reproduction.

Another reason for the high significance rating of arsenic is attributable to the unavailability of portable water in several rural communities in Ghana for which reason residents of these communities are dependent on raw water for drinking purposes. The residents could end up ingestion arsenic, which is abundantly present in the form arsenopyrite in several gold mining communities in Ghana making arsenic available for ingesting through drinking water. The significantly high concentration of arsenic in sampled water bodies in Ghana could have dire consequences on some provision and cultural ecosystem services including the availability of raw water to be treated for drinking purposes and water for recreational purposes respectively (Table 4, Table 5). Arsenic was however given a low impact score (Table 4) for arsenic contaminated water as a result of galamsey used for irrigation because plants uptake arsenic from soil in two main forms namely arsenate and arsenous acid. On entering the root cells, arsenate is quickly reduced to arsenite and channelled into media external to the plants or transported to shoots. A considerable number of plant species are known to be arsenic excluders and are unresponsive to arsenic over a wide range of concentrations in soils for which reason the concentration of arsenic in plants usually is very low [[51], [52], [53], [54]].

The significantly high concentration of mercury in water bodies in comparison to the permissible levels P < 0.0001 (Table 3) has a very high significance rating based on the quantitative, defensible impact characterization (Table 3). This is because the mercury concentration exceeded the permissible levels in water bodies affected by galamsey activities in Ghana over 87,000 times (Table 2). Elemental mercury in effluent generated by galamsey activities could be converted to methylmercury by microorganisms and absorbed by phytoplankton making it available for accumulation in consumers in the food chains linked to filter feeders and sediments of aquatic systems in Ghana. Mercury a known toxic heavy metal will have a very high significance rating because the methylmercury species of mercury is among the micropollutants that are most bioaccumulated and environmentally persistent. The bioaccumulation of methylmercury in fish and its subsequent biomagnification is known to be of risk to humans [55,56]. The transport of mercury vapour released during the process of amalgamation and gold recovery by galamsey miners, and its subsequent inhalation has the potential of seriously impairing the excretory functions of the kidneys, the transfer of impulses by the nervous system and the cognitive functions of the brain of humans. This is yet another reason why the emission of mercury due to galamsey activities received a very high significance rating (Table 4, Table 5, Table 6) [57,58].

Turbidity levels of galamsey impacted rivers in Ghana can exceed the permissible turbidity levels for surface water by as much as 1600 times (Table 2). This significantly high turbidity levels P < 0.0001 (Table 2) could reduce the sunlight intensity reaching aquatic plants affecting photosynthesis with subsequent impacts on dissolved oxygen levels and the growth of aquatic plants crucial for primary production in aquatic ecosystems for which reason its impact is rated high [18,20,59]. Another reason for the high impact significance rating of suspended solids is attributed to the huge volumes of slurry and mud, which constitute the main suspended solids, generated during alluvia galamsey gold mining activities including abstraction and sluicing which significantly increases turbidity in water bodies in Ghana. The slurry and mud, which are predominantly made of silt, sand and clay, darken water bodies, disrupting the migration of aquatic organisms that rely on vision for courtship and spawning. Additionally high turbidity because of suspended solids could lead to clogging of gills of fish, settling in cracks/crevices, impairment of the hunting ability of predatory aquatic organisms, affect food chains and ultimately protein sources of human beings. Huge volumes of suspended solids with its associated high turbidity in galamsey affected water bodies in Ghana could affect the habitats of traditional and indigenous fish species with the functionality and integrity of ecosystems and their associated provision ecosystem services ultimately being threatened ([18,60]; Van Dorst et al., 2018; [23,43,61,62]). High turbidity in surface water bodies in Ghana could impact negatively on cultural ecosystem services (Table 5) not limited to recreation, tourism, aesthetic experience, spiritual, religious, educational and cultural heritage values. The significantly high levels of suspended solids (Table 3) could lead to browning of water bodies in Ghana which could stimulate a bloom of the algae *Gonyostomum semen* with a resultant decrease in primary production associated with food chains and food webs necessary for fish biomass much sort after by anglers. The *Gonyostomum semen* bloom could also cause allergic reactions and skin irritation to prospective swimmers who will subsequently avoid such water bodies depriving them of recreational value that can be obtained from water bodies [47,[63], [64], [65]].

## 8 Conclusions

The significant quantities of arsenic, mercury and suspended solids generated during galamsey activities in Ghana has diverted the flow of several rivers, altered light penetration, destroyed aquatic habitats, fragmented aquatic habitats, and destroyed aquatic ecosystems in Tontokrom in the Ashanti Region, Kyekyewere/Akropong in the Central Region and Tarkwa Nsuam in the Western Region leading to loss of aquatic biodiversity in Ghana. The silt, sand, and clay the main suspended solids generated during galamsey activities, has darkened water bodies in Ghana, clogged the gills of fish, reduced the spawning sites of fish, and reduced the hunting ability of predatory aquatic organisms in Ghana.

Provision services not limited to raw water to be treated for domestic purposes, raw water for irrigation, raw water for aquaculture, fish harvest, raw biotic material (algae for fertilizer), and ornamental resources have seen a significant reduction due to an upsurge in silt, sand, and clay the main suspended solids generated during galamsey activities in Ghana. Cultural services including knowledge associated with folklore, aesthetic, religious and spiritual values derived from rivers Pra, Desu, Oti and Offin in Ghana are being lost to huge quantities of silt, sand, and clay generated during galamsey activities. The self-cleansing ability, flood regulation ability and habitat structure maintenance of several rivers in Ghana has been significantly impacted by silt, sand, and clay the main suspended solids generated during galamsey activities.

The metalloid arsenic released during gold recovery by galamsey activities was rated high in significance due to most gold deposits in Ghana mainly occurring within mineralized precambrian greenstones with the gold trapped as arsenopyrite. The high significance rating of arsenic is attributed to hydrobionts toxicity in rivers Pra, Desu, Oti and Offin caused by the highly hazardous persistent inorganic form of arsenic released during galamsey which affects fish growth, fish behaviour, fish reproduction and depletes fish stocks an important provision service of these rivers.

The heavy metal mercury used by the galamseyers for amalgamation activities and had a very high significance rating based on the quantitative, defensible impact characterization due to the persistence of mercury in aquatic environments. The mercury which could be converted to methylmercury by microorganisms and absorbed by phytoplankton bioaccumulating and biomagnifying in fish, snails and crabs along aquatic food chains and food webs across the length and breadth of Ghana.

## CRediT authorship contribution statement

**Kenneth Bedu-Addo:** Writing – review & editing, Writing – original draft, Supervision, Software, Methodology, Investigation, Formal analysis, Conceptualization. **Louis Boansi Okofo:** Writing – review & editing, Software. **Augustine Ntiamoah:** Writing – review & editing, Software, Formal analysis. **Henry Mensah:** Writing – review & editing, Formal analysis.

## Recommendations

To actualize Ghana's push towards achieving SDG targets 6.1 and 6.6 of goal 6 which seeks to ensure ‘safe and affordable drinking water’ and ‘protect and restore water-related ecosystems’ respectively, galamsey activities should be done under a licenced regime devoid of aquatic pollution, destruction of ecosystems and their services through regular capacity building programme on sustainable mining techniques viz the use of mercury-free mineral processing equipment and through the implementation of the cycle of cause-effect-outlook relationship for responsible artisanal mining in Ghana as shown in Fig. 5.

## Data availability statement

Data sets generated during the current study are available from the corresponding author on reasonable request. The data sets were obtained mostly from the water sources which Ghana's water company abstracts its raw water which is under siege by artisanal miners and hence mostly demarcated as security zones.

## Dual publication

I declare that the results/data/figures in this manuscript have not been published elsewhere, nor are they under consideration (from you or one of your Contributing Authors) by another publisher.

## Authorship

The corresponding author has read the Springer journal policies on author responsibilities (opens in a new window) and submits this manuscript in accordance with those policies.

## Third party material

I declare that all the material is owned by the authors and/or no permissions are required.

“All authors have read, understood, and have complied as applicable with the statement on "Ethical responsibilities of Authors" as found in the Instructions for Authors and are aware that with minor exceptions, no changes can be made to authorship once the paper is submitted.”

## Funding declaration

None.

## Declaration of competing interest

The authors declare that they have no known competing financial interests or personal relationships that could have appeared to influence the work reported in this paper.
